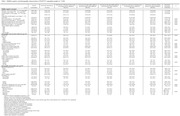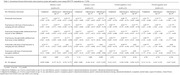# Life‐course food insecurity is associated with cognition in later life: Evidence from the National Longitudinal Youth Survey 1979 Cohort

**DOI:** 10.1002/alz.090144

**Published:** 2025-01-09

**Authors:** Erin E Esaryk, Anusha M Vable, Jillian Hebert, Lucia Pacca, S. Amina Gaye, Aayush Khadka, Sonali Singh, Whitney M Wells, Tanisha G Hill‐Jarrett, Suzanna Martinez

**Affiliations:** ^1^ University of California, San Francisco, San Francisco, CA USA; ^2^ University of California San Francisco, San Francisco, CA USA; ^3^ University of California, San Francisco, 94158, CA USA

## Abstract

**Background:**

Life‐course food insecurity may influence cognition through pathways of stress and poor health. Yet, few longitudinal studies examine whether life‐course food insecurity is associated with cognition among U.S. adults.

**Methods:**

We performed a secondary data analysis using National Longitudinal Youth Survey 1979 data from 1979‐2016 (N = 7,320). Annual food security status was estimated between the ages of 22‐45 using 2008 Supplemental Nutrition Assistance Program eligibility requirements for household income and assets as a proxy. Sequence and cluster analyses were used to identify prototypical food security status trajectories. We identified seven trajectory groups ranging from *persistently food secure* to *persistently food insecure*. Trajectory groups varied by order, timing and duration of food security status. Cognition (ages 45‐59) was measured using five assessments, which were summarized into z‐scores (attention, memory, overall cognitive) and Langa‐Weir cognition score. We used linear regression to model associations between food security trajectory groups and cognition, while controlling for covariates.

**Results:**

Most respondents were in the *persistently food secure* group (65%), with a Langa‐Weir cognition score of 15.84 (SD = 4.57, scale = 0‐27), and z‐scores as follows: memory, 0.00 (SD = 0.00); attention, 0.01 (SD = 0.76), overall cognitive, 0.00 (SD = 0.00). Compared to the *persistently food secure* group, the following groups were negatively associated with cognition after adjustment: *persistently food insecure* (β Langa‐Weir cognition: ‐2.08, 95% CI [‐2.64,‐1.52]), *food insecure with bouts of food security in emerging and early adulthood* (β Langa‐Weir cognition: ‐1.51,[‐2.19, ‐0.83]), *food secure through middle adulthood and food insecure in later adulthood*, (β Langa‐Weir cognition: ‐1.19, [‐1.73, ‐0.65]), and *food insecure through middle adulthood and food secure in later adulthood* (β Langa‐Weir cognition: ‐0.66, [‐1.17, ‐0.14]) Coefficients were consistent for z‐score outcomes.

**Conclusions:**

We observed a dose‐response trend, such that cognitive ability decreased among groups with prolonged food insecurity exposure. Food insecurity is modifiable; therefore, strengthening policies to improve food access among Americans is imperative.